# Supplementary material for: Can the home experience in luxury hotels promote pro-environmental behavior among guests?
Source: PLoS One. 2023 Dec 28;18(12):e0284981. doi: 10.1371/journal.pone.0284981 (PMC10754442; doi:10.1371/journal.pone.0284981)
Supplement: Attachment — Submitted filename: Respond to Reviewer comments.docx [file pone.0284981.s002.docx]

| Reviewer comments: | Comments addressed: |
| --- | --- |
| **Reviewer 1** |  |
| The contribution of the study should be better highlighted in the introduction section. | Thank you very much for your feedback. In the introduction section, we have described the contribution of this study in more specific terms.  Please see details **on page 3-4 with highlighted text.** |
| I believe that the LR could have been more critical in nature. | Thank you for the constructive suggestion. We have added a more critical discussion of luxury hotels.  Please see the detailed explanation highlighted on **page 2.** |
| Figure 1: the conceptual model requires more discussions and justifications. | Thank you for pointing this out. We have added a more specific explanation to this conceptual model.  Please see details **on page 7 with highlighted text.** |
| The Moderating Effect of PEBP requires additional theoretical discussions. | Thank you for your suggestion. We have added a theoretical exploration of the moderating effect of PEBP, thus making the hypothesis more convincing.  Please see details **on page 6-7 with highlighted text.** |
| Maybe the author could discuss more on the limitation of the methods used (both the Qualitative and the Quan). | Thank you for pointing this out. In the research methods section, we have added further discussion on the limitations of qualitative and quantitative research and the reasons for using a mixed research approach in this study.  Please see the **first paragraph on page 8** for specific details **with highlighted text.** |
| the practical and theoretical implications of the study would benefit from more discussion. | Thank you for the constructive suggestion. We have revised the theoretical and practical implications section, especially emphasizing the theoretical implications, which we hope will lead to a more in-depth discussion of this study.  Please see details **on page 16-17 with highlighted text.** |
| to cross check typos and references. | Thank you for your suggestion. We apologize for any inconvenience to your review, and we have double-checked for typos and references. |

| Reviewer comments: | Comments addressed: |
| --- | --- |
| **Reviewer 2** |  |
| 1. in the abstract, present the results found in a more objective and clear way; | Thank you very much for your feedback. We have removed any redundant sentences to make the summary more concise.  Please see details **on page 2 with highlighted text.** |
| 2. in the introduction and literature review sections, explain the concept of luxury hotel and explain the option for this type of hotel, as one of the key words of the paper is "luxury hotel" and this reference is made in the title of the article. The words "luxury hotel" first appear in the manuscript at line 245 in research methodology. | Thank you for your suggestion. In the introduction section, we have added the concept of luxury hotels and a critical discussion of their roles.  Please see details **on page 2 with highlighted text.** |
| 3. on line 230 and 231, author said "In this study, mixed methods were used, integrating qualitative and quantitative data to gain a deeper understanding of the research questions". However, this manuscript does not have research questions. | Thank you for pointing this out. Regarding "In this study, mixed methods were used, integrating qualitative and quantitative data to gain a deeper understanding of the research questions"，our intention was to explain the reasons for adopting a mixed research approach in this paper, and to clarify the limitations of using qualitative and quantitative research.  In addition, the core research question of this paper is "whether the home experience has an impact on the pro-environmental behavior of luxury hotel customers", and the choice of research method is specifically meant to address this question.  Please see the **first paragraph on page 8** for specific details **with highlighted text.** |
| 4. in research methodology section a more detailed explanation about the option to carry out a single focus group included six hotel guests + three professors in tourism management + two hotel managers would be necessary, as well as the duration (minutes) of this focus group and the way in which data were recorded (audio?). | Thank you for your suggestion. We have added the member presentations and data acquisition process for the focus group discussions.  Please see the **second paragraph on page 8** for specific details **with highlighted text.** |
| 5. at the end of analysis of results section, a comparison with results of studies from other academic literature will be necessary. | Thank you for your constructive suggestion. We have revised the conclusion by adding a dialog discussion of the existing relevant literature.  Please see details **on page 15-16 with highlighted text.** |
